# Cofilin activation in peripheral CD4 T cells of HIV-1 infected patients: a pilot study

**DOI:** 10.1186/1742-4690-5-95

**Published:** 2008-10-17

**Authors:** Yuntao Wu, Alyson Yoder, Dongyang Yu, Weifeng Wang, Juan Liu, Tracey Barrett, David Wheeler, Karen Schlauch

**Affiliations:** 1 Department of Molecular and Microbiology, George Mason University, Manassas, VA, 20110, USA; 2 Clinical Alliance For Research & Education – Infectious Diseases, LLC, Annandale, VA, 22003, USA; 3 Department of Genetics and Genomics, Boston University School of Medicine, Boston, MA, 02118, USA

## Abstract

Cofilin is an actin-depolymerizing factor that regulates actin dynamics critical for T cell migration and T cell activation. In unstimulated resting CD4 T cells, cofilin exists largely as a phosphorylated inactive form. Previously, we demonstrated that during HIV-1 infection of resting CD4 T cells, the viral envelope-CXCR4 signaling activates cofilin to overcome the static cortical actin restriction. In this pilot study, we have extended this *in vitro *observation and examined cofilin phosphorylation in resting CD4 T cells purified from the peripheral blood of HIV-1-infected patients. Here, we report that the resting T cells from infected patients carry significantly higher levels of active cofilin, suggesting that these resting cells have been primed *in vivo *in cofilin activity to facilitate HIV-1 infection. HIV-1-mediated aberrant activation of cofilin may also lead to abnormalities in T cell migration and activation that could contribute to viral pathogenesis.

## Findings

Cofilin is a member of the actin-depolymerizing factor (ADF) family of proteins [1] that play a central role in regulating actin dynamics [2,3]. The actin-severing and depolymerization activities of cofilin are essential in controlling cell polarity [4], cell motility [5] and cell division [6,7]. In the human immune system, cofilin has also been implicated in two hallmark activities of T cells, namely chemotaxis and T cell activation [8]. In chemotaxis, directed cell movement towards chemoattractants is controlled by localized cortical actin polymerization and depolymerization, and cofilin is the driving force for promoting the cortical actin dynamics [9]. In antigen-specific T cell activation the reorganization of the cortical actin plays a critical role in the formation of the immunological synapse. Engagement of CD2 or CD28 receptors but not TCR results in cofilin activation and its association with the actin cytoskeleton [10]. Peptides that block cofilin binding to actin result in severe defects in T cell activation [11].

Cofilin activity is regulated through phosphorylation and dephosphorylation at serine-3 by the simultaneous actions of cofilin kinases and phosphatases [12-14]. Phosphorylated cofilin is unable to bind to F-actin; thus cofilin is inactivated by phosphorylation and activated by dephosphorylation [13,14]. The direct upstream kinases that inactivate cofilin are the LIM kinases (LIMK1 and LIMK2) [15,16], whereas several serine phosphatases such as slingshot, chronophin [17,18], PP1α and PP2A [19] dephosphorylate and activate cofilin.

Recently, we [20] and others [21] have demonstrated that in unstimulated resting CD4 T cells purified from the peripheral blood, cofilin exists largely as the phosphorylated form, implying that in the absence of chemotactic stimulation or T cell activation, cofilin is largely inactive. We have also suggested that this restricted cofilin activity in resting T cells inhibits the cortical actin dynamics, hindering viral post-entry migration. Thus, HIV-1 hijacks chemokine receptor signalling through CXCR4 to trigger the activation of cofilin. This process increases the cortical actin dynamics, facilitating viral nuclear migration [20,22].

**Figure 1:**
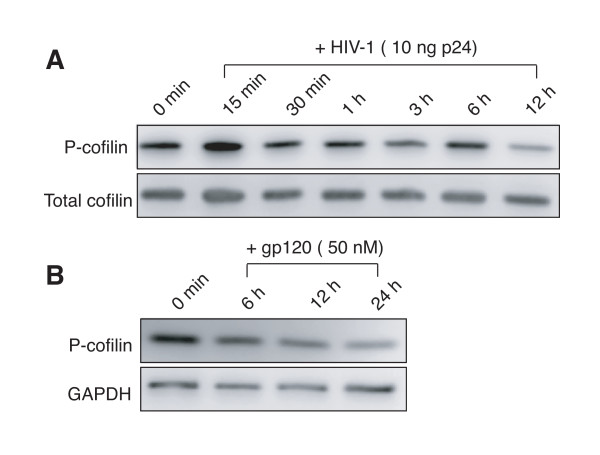
**Activation of cofilin in resting CD4 T cells cultured *in vitro *and stimulated with HIV-1 and gp120**. Resting CD4 T cells were purified from uninfected donors by antibody-mediated negative depletion using Dynalbeads as previously described [20]. Cells were cultured overnight in the absence of cytokines or activation, and then stimulated with HIV-1 (10 ng p24) (A) or with gp120 (50 nM) for various times at 37°C as indicated. Stimulated cells were lysed and analyzed by western blot using antibodies against the phosphorylated cofilin (P-cofilin) or total cofilin or GAPDH for controls.

Given the fact that in infected patients, CD4 T cells are chronically exposed to gp120, we decided to investigate the outcome of persistent gp120 stimulation on cofilin phosphorylation. To address this question, we initially used resting CD4 T cells purified from HIV negative donors and stimulated them with HIV-1 or gp120 for extended periods of time. We incubated cells with the virus for several hours up to 24 hours instead of minutes. Persistent stimulation of a receptor has been known to affect down-stream targets differently than transient stimulation [23]. As shown in Figure 1A, we observed cycles of cofilin phosphorylation and dephosphorylation with the prolonged treatment. These data suggest that persistent stimulation with HIV will likely have a lasting impact on the cofilin activity. We also repeated this experiment using purified gp120 and observed persistent cofilin dephosphorylation (Figure 1B). The variation in cofilin responses between HIV particles and gp120 may be related to differences in dosage or gp120 conformation. HIV particles carry the gp120 trimer on the surface, whereas the purified gp120 protein we used is a monomer. This is reminiscent of the CD40 ligand (CD40L) in its distinctive signalling properties as a trimer or as a monomer [24]. The strength and persistence of CD40L stimulation dictate the capacity of dendritic cells either to migrate to draining lymph nodes or to secrete locally inflammatory cytokines [24].

**Figure 2:**
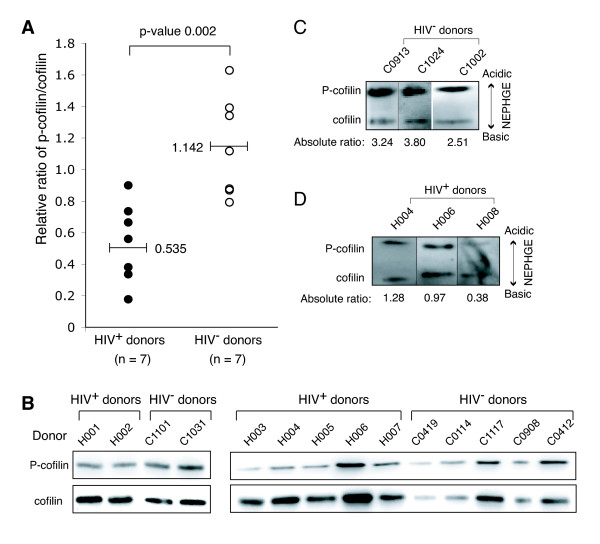
**Activation of cofilin in resting CD4 T cells of HIV-1-infected patients**. (A) Resting CD4 T cells were purified by antibody-mediated negative depletion using Dynalbeads. Cells purified from HIV-1-infected and uninfected donors were cultured overnight in the absence of cytokines or activation, and then lysed and analyzed by western blot using antibodies against P-cofilin or total cofilin. The relative ratios of P-cofilin to total cofilin were measured and plotted. HIV-1-infected patients had statistically-significant lower ratios of P-cofilin/cofilin (0.535 versus 1.142, *p *= *0.002*), suggesting higher levels of active cofilin. For statistic analysis, a two-tailed Student's t-test on the means resulted in a p-value of *p *= *0.002*. At a pre-determined significance level of 0.05, this shows that the difference in the mean ratios of the two sample groups is statistically significant. A standard power computation showed that the t-test was very well powered (95.2%) for this study. (B) Shown are the longer exposures of the western blots used in (A). The results were confirmed by NEPHGE-western blot to directly separate P-cofilin to active cofilin, and then probed with an anti-cofilin antibody [20]. Shown are the absolute ratios of P-cofilin to active cofilin in HIV-1 negative donors (C) and HIV-1-infected donors (D).

**Figure 3:**
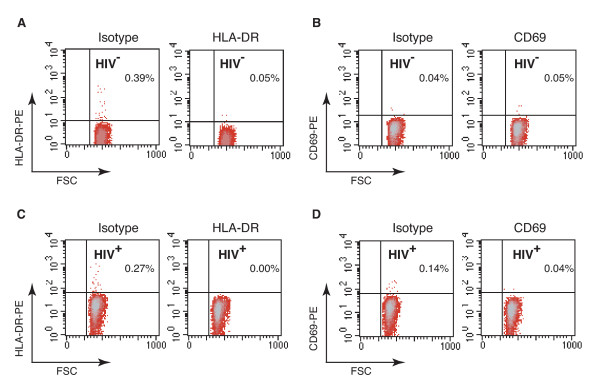
**Lack of detection of T cell activation markers on the surface of resting CD4 T cells from HIV-1-infected and uninfected donors**. Resting CD4 T cells were purified by antibody-mediated negative depletion using Dynalbeads. Cells purified from HIV-1-infected and uninfected donors were cultured overnight in the absence of cytokines or activation, and then stained for surface expression of HLA-DR or CD69. Shown are flow cytometry analyses of cells stained with a PE-labelled anti-human HLA-DR antibody (A and C, right panel) or a similarly labelled isotype control antibody (A and C, left panel). Cells were also stained with a PE-labelled anti-human CD69 antibody (B and D, right panel) or a similarly labelled isotype control antibody (B and D, left panel). Cells from the HIV negative donor (HIV^-^) were used in (A) and (B), and cells from the HIV-1-infected donor (HIV^+^) were used in (C) and (D).

Based on these results, we also hypothesized that activation of cofilin may also occur in the resting CD4 T cells of HIV-1-infected patients, considering that these resting T cells are chronically exposed to gp120 during the course of infection, and that even in patients on HAART, latently infected cells persist and low levels of viral replication take place [25-27]. Additionally, the threshold for coreceptor activation of signalling has been shown to be as few as two HIV virion particles [28]. Thus, we set up a small-scale pilot study to probe cofilin activity in resting cells purified from HIV-1-infected and uninfected subjects. Peripheral blood resting CD4 T cells from eight infected (Additional file 1) and ten uninfected subjects were purified by negative depletion, unstimulated and then analyzed by immunoblotting for both phospho-cofilin and total cofilin. As shown in Figure 2, in the resting CD4 T cells of uninfected subjects, cofilin exists primarily in its inactive phosphorylated form in the absence of chemotactic stimulation or T cell activation [20,21] (Figure 2C). In contrast, in HIV positive patients, significantly lower ratios of phospho-cofilin to total cofilin (HIV^-^, 1.142; HIV^+^, 0.535; *p = 0.002*) (Figures 2A and Figure 2B) were observed, suggesting a significant shift towards cofilin activation. These results were further confirmed by NEPHGE-western blot to measure the absolute ratio of phospho-cofilin to active cofilin (Figures 2C, Figure 2D). Again, we observed considerably lower ratios of phospho-cofilin to active cofilin in HIV-1-infected patients, confirming the upregulation of cofilin activity in resting CD4 T cells of HIV-1-infected patients. Given the great extent of cofilin activation and the fact that a majority of resting CD4 T cells in patients are not infected (0.2–16.4 HIV-latently infected cells per 10^6 ^resting CD4 T cells [29]), these data imply a global activation of cofilin in resting CD4 T cells, not just those infected by HIV-1. Therefore, indirect mechanisms, such as contact with viral or cell-free gp120 or chronic immune activation, may be responsible for the activation of cofilin in resting CD4 T cells in patients. Importantly, the cofilin activation observed is not a result of general T cell activation, since the population of CD4 T cells purified from patients is quiescent, judged by the lack of activation markers such as HLA-DR or CD69 on the cell surface (Figure 3), similar to a previous observation [30]. This also appears to be consistent with our previous demonstration that although stimulation with gp120 can trigger cofilin activation, it does not activate resting T cells [20]. Our data also demonstrate that in the peripheral blood of infected patients, resting CD4 T cells have largely been altered or primed, at least in cofilin activity, to facilitate HIV-1 infection. Nevertheless, the small patient population as well as lack of multiple controls and long-term follow up studies did not permit us to conclude that the cofilin activation observed is necessarily the result of chronic gp120 exposure, although gp120 has a demonstrated ability to trigger cofilin activation *in vitro *[20]. Further large-scale studies are needed to address this correlation and other critical questions such as the possible relationship between cofilin activation and disease progression. Future studies are also required to determine whether the status of cofilin correlates with drug treatment. Our previous *in vitro *study [20] and this small-scale pilot investigation certainly serve as a rationale for future clinical studies.

HIV-1-mediated aberrant activation of cofilin in resting CD4 T cells may affect normal T cell migration and T cell activation. In the human immune system, cofilin is directly involved in chemotaxis and T cell activation. For example, cofilin was shown to affect SDF1α-driven T cell chemotaxis, and blocking cofilin phosphorylation diminishes actin reorganization and normal chemotactic response [9]. During T cell activation, cofilin is activated by co-stimulation signals to mediate cortical actin reorganization, which plays a critical role in the formation and stabilization of the immunological synapse. It is commonly known that genetic defects affecting actin activity by means of a deficiency in signaling molecules, such as WASP, cause immunodeficiency [31,32]. It would not be a surprise if cofilin dysregulation also results in T cell-mediated immunodeficiencies, given the central role of cofilin in regulating actin dynamics in T cells [33].

The demonstration of cofilin activation in resting CD4 T cells of HIV-1-infected patients offers new avenues for investigation into viral pathogenesis. It has long been recognized that the residual CD4 T cells in HIV-1-infected patients have numerous functional abnormalities, such as loss of T helper function [34], T cell anergy [35,36], increased T cell proliferation [37] and abnormal T cell homing and migration [38,39]. These T cell defects largely result from a bystander effect [40]. It remains to be determined whether some of these abnormalities are directly linked to aberrant activation of cofilin in resting CD4 T cells. Additionally, as shown in this pilot study, the peripheral CD4 T cells in HIV-1 patients strikingly resemble the migratory T lymphoma cells in terms of carrying active cofilin [21,41]. It is likely that these CD4 T cells also have abnormal migratory behaviours associated with aberrant cofilin activation. It remains unknown whether migratory abnormalities could contribute to the eventual destruction of T cells in lymph nodes or tissues. Finally, the identification of cofilin as a critical molecule in resting CD4 T cells of infected patients may serve as a diagnostic marker to reflect alterations of T cell function in disease progression.

## Abbreviations

LIMK1: LIM Domain Kinase 1; TCR: T Cell Receptor; HAART: Highly Active Antiretroviral Therapy; NEPHGE: Nonequilibrium pH Gel Electrophoresis; HLA-DR: Human Leukocyte Antigen-DR; WASP: Wiskott-Aldrich Syndrome Protein; SDF1α: Stromal-Cell-Derived Factor 1α.

## Competing interests

The authors declare that they have no competing interests.

## Authors' contributions

YW conceived of the study, supervised blood donation, performed T cell purification and wrote the manuscript. AY, DY, WW, and JL performed cell purification, western blot, and analysis. TB and DW supervised HIV+ donor recruiting and testing. KS performed statistical analyses.

## Supplementary Material

Additional file 1 Click here for file
