# Supplementary material for: Cofilin activation in peripheral CD4 T cells of HIV-1 infected patients: a pilot study
Source: Retrovirology. 2008 Oct 17;5:95. doi: 10.1186/1742-4690-5-95 (PMC2576353; doi:10.1186/1742-4690-5-95)
Supplement: Additional file 1 [file 1742-4690-5-95-S1.pdf]

**Additional file 1.**

## HIV-1-infected donor information

| HIV+ Donor | CD4 count (cells / µl) | Viral RNA (copies / ml) | Drug Treatment at the Time of Blood Draw | Notes |
|---|---|---|---|---|
| 001 | 270 | 50 | Truvada, Norvir, Reyataz | Treated for 12 months. |
| 002 | 328 | 50 | No Treatment | Treated with Truvada, Norvir, Reyataz for 11 months, and discontinued 28 days before the blood draw. |
| 003 | 140 | 1,310 | Ziagen, Videx EC, Viread, Kaletra | Treated for 3 years with various RT and protease inhibitors. The patent showed signs of drug resistance. |
| 004 | 191 | 50 | Combivir, Viread, Kaletra, Maraviroc | Treated for 19 months. |
| 005 | 3.8 | 65,100 | Videx EC, Maraviroc, Truvada | Treated with Videx EC and Maraviroc for 10 months, Truvada for 7 months. The patient showed signs of drug resistance. |
| 006 | 465 | 84 | AZT, Lexiva, Norvir | Treated with Lexiva, Norvir for 7 months, AZT for 11 months. |
| 007 | 3 | 280,000 | GS-9137, Norvir | Treated for 6.5 months. The patient has been treated with 16 different drugs dated back to 1999, with numerous treatment interruptions. The patient also showed signs of drug resistance. |
| 008 | 229 | 50 | Kaletra, Truvada, Fuzeon, Maraviroc/Placebo ? | Treated for 10 months. The patient participated in a Maraviroc clinical trial that is still ongoing. Drug status remains unknown. |

* All HIV-1 negative donors have CD4 T cell counts above 1,000 cells / µl.
